# Circ_0004104 participates in the regulation of ox-LDL-induced endothelial cells injury via miR-942-5p/ROCK2 axis

**DOI:** 10.1186/s12872-022-02959-1

**Published:** 2022-12-02

**Authors:** Yuanyuan Zhang, Shaojun Wang, Sicong Guo, Xinzhong Zhang, Chuan Yang, Guangsheng Su, Jiye Wan

**Affiliations:** Cardiovascular Internal Medicine Department, Shengjing Hospital of China Medical University, No. 36, Sanhao Street, Heping District, Shenyang, 110000 China

**Keywords:** Circ_0004104, miR-942-5p, ROCK2, Atherosclerosis, HUVECs

## Abstract

**Background:**

Cardiovascular disease was the most common disease among the elderly with high morbidity and mortality. Circ_0004104 was demonstrated to be involved in the regulation of atherosclerosis.

**Methods:**

Quantitative real-time polymerase chain reaction was employed to measure the expression of circ_0004104, miR-942-5p and Rho associated coiled-coil containing protein kinase 2 (ROCK2). Cell proliferation was tested by 3-(4,5-dimethyl-2-thiazolyl)-2,5-diphenyl-2-H-tetrazolium bromide (MTT) assay. Cell apoptosis was measured by flow cytometry, and tube formation assay was used to detect the angiogenesis ability of cells. Western blot assay was performed to assess protein levels. Enzyme‑linked immunosorbent assay was used to detect the release of IL-1β and TNF-α. The relationship between miR-942-5p and circ_0004104 or ROCK2 was identified by dual-luciferase reporter assay, RNA immunoprecipitation (RIP) assay, and RNA pull-down assay.

**Results:**

Oxidized low-density lipoprotein (ox-LDL) inhibited the proliferation of human umbilical vein endothelial cells (HUVECs) and promoted apoptosis in a dose-dependent manner. Circ_0004104 was increased in serum of atherosclerosis patients and ox-LDL-treated HUVECs, and silence of circ_0004104 promoted the proliferation of ox-LDL-exposed HUVECs and inhibited cell apoptosis. MiR-942-5p downregulation reversed si-circ_0004104-mediated influences in HUVECs upon ox-LDL exposure. ROCK2 was the target of miR-942-5p and circ_0004104 regulated the expression of ROCK2 through sponging miR-942-5p. ROCK2 abated the influences of miR-942-5p in ox-LDL-stimulated HUVECs. Circ_0004104 was increased in the exosomes derived from ox-LDL-exposed HUVECs, and the expression of circ_0004104 was promoted in HUVECs after stimulation with ox-LDL-treated HUVECs cells-derived exosomes.

**Conclusion:**

Circ_0004104 downregulation receded ox-LDL-induced injury in HUVECs through miR-942-5p and ROCK2.

**Supplementary Information:**

The online version contains supplementary material available at 10.1186/s12872-022-02959-1.

## Background

Studies have found that many countries are entering or have entered an aging society, and cardiovascular diseases are common diseases among the elderly with high morbidity and mortality [1]. Atherosclerosis is the most important common vascular disease. Hypertension, diabetes, hyperlipidemia, and other diseases are also prone to induce atherosclerosis [2]. Therefore, the pathogenesis and clinical diagnosis of atherosclerosis are attracting more and more attention from doctors, so it is particularly important to explore new therapeutic targets for atherosclerosis.

A large number of testimonies exhibit that circRNAs regulate the occurrence of atherosclerosis by regulating mRNA expression through miRNA sponge interaction [3]. Zhang et al. [4] found that circ_0003204 was obviously increased in oxidized low-density lipoprotein (ox-LDL)-induced human aorta endothelial cells, and its knockdown inhibited cell progression through miR-370 and TGFβR2. Circ-Sirt1/miR-132/212/SIRT1 and circ_0010283/miR-370-3p/HMGB1 have been shown to regulate the progression of vascular smooth muscle cells (VSMCs) [5, 6]. Circ_0000231 participated in ox-LDL-induced human umbilical vein endothelial cells (HUVECs) damage through miR-135a-5p and CLIC4 [7]. Circ_0004104 has been shown to be a biological target for coronary artery disease[8], and gastric cancer[9] and has also been shown to be involved in the progression of cardiovascular disease [10, 11]. However, the mechanism of circ_0004104 regulation of atherosclerosis is not completely clear.

MiRNAs have been reported to influence the development of atherosclerosis by regulating endothelial and smooth muscle cell development [12, 13]. MiR-21-3p might promote the proliferation and migration of VSMCs through PTEN, thereby accelerating the process of atherosclerosis [14]. MiR-342-5p regulates H_2_O_2_-damaged HUVECs cell apoptosis by inhibiting PP1R12B [15]. MiR-942-5p has been certified to be involved in the progression of oral squamous cell carcinoma [16], ovarian cancer [17], cervical cancer [18], and atherosclerosis [19]. However, the relationship and role of miR-942-5p and circ_0004104 in atherosclerosis remain unclear, here we sought to explore these.

MiRNAs function in organisms by degrading or inhibiting mRNA expression of target genes[20]. Previous research has indicated that Rho associated coiled-coil containing protein kinase 2 (ROCK2) is one of the isoforms of the ROCK family [21]. A series of recent studies demonstrated that ROCKs are substantially implicated in cardiovascular diseases, containing atherosclerosis [22, 23]. Meanwhile, the dysregulation of ROCKs might partake in the regulation of various cardiovascular pathological processes [21, 24]. Apart from that, related literature has confirmed that ROCK2 is boosted in atherosclerosis tissues and HUVECs cells, and restored the promotion effects of miR-135a-5p on ox-LDL-exposed HUVECs [23]. But, the function of miR-942-5p, ROCK2, and circ_0004104 in atherosclerosis has not been reported.

This study is the first to confirm the role of the circ_0004104/miR-942-5p/ ROCK2 axis in atherosclerosis, providing a new possibility for the treatment of atherosclerosis.

## Materials and methods

### Patients and cells

Blood samples were collected from 21 atherosclerosis patients and 17 healthy volunteers from Shengjing Hospital of China Medical University to prepare serum samples, and the participants provided informed consent. All examined samples were stored at − 80 °C. This research was done under the Ethics Committee in Shengjing Hospital of China Medical University and was carried out according to the guidelines of the Declaration of Helsinki.

HUVECs were furnished by American Type Culture Collection (Manassas, VA, USA), and grown in Dulbecco’s modified Eagle’s medium (DMEM; cat.no. SG 12,100; Solarbio, Beijing, China) with 5% CO_2_ at 37 °C_._ 10% fetal bovine serum (FBS; cat.no.10082; Invitrogen, Paisley Scotland, UK) and 1% penicillin–streptomycin (cat. no. 15140–122; Invitrogen) were added to DMEM. To establish Atherosclerosis models, HUVECs were treated with 0 μg/mL, 25 μg/mL, 50 μg/mL and 100 μg/mL of ox-LDL (cat. no. H7950; Solarbio, Beijing, China) for 24 h.

### Quantitative real-time polymerase chain reaction (qRT-PCR)

Total RNA in cells and serum samples was extracted with the help of Trizol (cat.no.15596018; Thermo Fisher Scientific, Waltham, MA, USA), followed by incubation with RNase R (4 U/μg, cat. no. RNR07250; Epicentre, Illumina, San Diego, CA, USA) at 37 °C for 1 h. Then, cDNA was synthesized and amplified in PCRmax Alpha gradient PCR instrument (Staffordshire, UK) with SYBR Green PCR kit (cat. no. DRR820A; TaKaRa, Beijing, China). Relative RNA expression was normalized to GAPDH or U6, and calculated by the 2^–ΔΔCt^ method. All primers were obtained from Beijing Genomics Institute (BGI, Shenzhen, China), as shown in Table 1.

**Table 1:** Primers sequences used for PCR

Name	Primers (5′-3′)	
circ_0004104	Forward	AGACCTGTGACCTGGACAATG
	Reverse	GTGCACTTTGTGGCAAAGAA
ROCK2	Forward	TCCCGATAACCACCCCTCTT
	Reverse	CCTTGTGACGAACCAACTGC
miR-942-5p	Forward	GTATGATCTTCTCTGTTTTG
	Reverse	TGGTGTCGTGGAGTCGT
GAPDH	Forward	AAGGCTGTGGGCAAGGTCATC
	Reverse	GCGTCAAAGGTGGAGGAGTGG
U6	Forward	CTCGCTTCGGCAGCACATA
	Reverse	CGAATTTGCGTGTCATCCT

### Cell proliferation

The impacts of ox-LDL and circ_0004104 on cell proliferation were evaluated by 3-(4,5-dimethyl-2-thiazolyl)-2,5-diphenyl-2-H-tetrazolium bromide (MTT). Shortly, 0.5 g MTT (cat. no. M1020; Solarbio) was dissolved in phosphoric acid buffer (PBS; cat.no. 10010023; Thermo Fisher Scientific) of 100 mL and configured into 5 mg/mL MTT working solution. MTT was used to treat cells for 4 h, and then cells were added with dimethyl sulfoxide (DMSO; cat. no. D8371; Solarbio). OD value at 490 nm was detected by a microplate analyzer (Molecular Devices, Silicon Valley, California, USA).

### Cell apoptosis assay

Flow cytometry and Annexin V-FITC/PI apoptosis detection kit (cat.no. CA1020; Solarbio) were used to estimate HUVECs apoptosis. Briefly, ox-LDL-treated HUVECS were incubated with 5 μL Annexin V-FITC and PI and mixed with 400 μL binding buffer. Then apoptosis cells and normal cells were distinguished by FACS CantoII flow cytometry (BD Biosciences, San Jose, CA, USA) with BD FACSDiva software.

### Angiogenesis experiment

HUVECs were grown with serum-free medium for 16 h in advance, then the cells were inoculated into a 24-well plate containing Matrigel (cat. no. 354234; BD Biosciences) and cultured in serum-containing medium. The tube formation was observed under a microscope and photographed under the inverted microscope. The amounts of the formed tubes represented the tube forming capability of HUVECs.

### Western blot assay

Proteins that were extracted from ox-LDL-treated cells were separated with Sodium dodecyl sulfate–polyacrylamide gel electrophoresis (cat. no. P1200; SDS-PAGE, Solarbio) and polyvinylidene difluoride (PVDF) membrane (Amersham, Munich, Germany), and then PVDF membrane was blocked with TBST buffer containing 5% bovine serum albumin. The protein bands were incubated with primary antibodies: CyclinD1 (ab16663, 1:1000, Abcam, Cambridge, MA, USA), cleaved-caspase 3 (ab2302, 1:1000, Abcam), ROCK2 (ab125025, 1:1000, Abcam), GAPDH (ab9485, 1:1000, Abcam), and the secondary antibody (1:5000, ab205718, Abcam), respectively. Finally, the protein bands were incubated with ECL Western Blotting Substrate (cat. no. PE0010; Solarbio) and observed by a gel imaging analysis system. The original western blots were showed in Additional file 1.

### Enzyme‑linked immunosorbent assay (ELISA)

Cell supernatant of Atherosclerosis models was collected, and TNF-α (human) ELISA Kit (cat. no. 589201; Cayman Chemical, Ann Arbor, Michigan, USA) and IL-1β (human) ELISA Kit (cat. no. BMS224HS; eBioscience, San Diego, California, USA) were used to detect TNF-α and IL-1β contents in the supernatant, respectively.

### Cell transfection

Geneseed (Guangzhou, China) provided the stable circ_0004104 overexpression vector (pCD5-circ_0004104, circ_0004104) and the negative control (pCD5-ciR). Circ_0004104 specific small interfering RNA (si-circ_0004104), si-NC, miR-942-5p mimics (miR-942-5p), miR-NC, anti-miR-942-5p, anti-miR-NC, pcDNA-ROCK2 (ROCK2) and pcDNA were constructed by Sangon Biotech (Shanghai, China), followed by transfection into HUVECs using Lipo3000 reagent (cat. no. L3000015; Invitrogen).

### Dual-luciferase reporter assay

The forecasted circ_0004104 and ROCK2 sequences that bound miR-942-5p and site-directed mutation sequences were cloned into psiCHECK2 plasmid (Promega, Madison, WI, USA) and expressed as WT-circ_0004104, WT-ROCK2 3’UTR, MUT-circ_0004104 and MUT-ROCK2 3’UTR. Then plasmids were co-transfected into HUVECs with miR-942-5p or miR-NC, respectively. After 24 h, the luciferase activities of plasmids were detected according to Dual-Luciferase Reporter Assay System (cat. no. E1910; Promega).

#### RNA immunoprecipitation (RIP) assay

The RIP experiment was implemented by taking advantage of the RNA Immunoprecipitation Kit (cat. no. 17–700; Sigma-Aldrich, St. Louis, MO, USA) and qRT-PCR. In short, HUVECs were lysed in complete RIP lysis buffer, followed by a mixture with RIP buffer containing magnetic beads conjugated with anti-Argonaute2 (Ago2, ab186733, 1:100, Abcam) antibody or normal mouse IgG (ab172730, 1:100, Abcam). Finally, beads were washed twice with PBS, and total RNA was isolated and subjected to qRT-PCR analysis of circ_0004104, miR-942-5p, and ROCK2.

#### RNA pull-down assay

For testing the interaction between miR-942-5p and circ_0004104 or ROCK2 in HUVECs, RNA pull-down assay was conducted. In short, HUVECs transfected with biotinylated- miR-942-5p (bio-miR-942-5p) and bio-miR-NC (GenePharma, Shanghai, China) were harvested and lysed. After that, magnetic beads (cat. no. 112-05D; Invitrogen) was utilized to mix with the harvested cell lysates, followed by RT-qPCR analysis.

#### Isolation and identification of exosomes

Ox-LDL treated and untreated HUVECs were centrifuged at 300 ×*g* for 10 min, the sediments were discarded and liquid supernatant was collected with a 0.22 µm filter mini-column (Sigma-Aldrich). The supernatant was centrifuged at 110,000 ×*g* for 70 min, the bottom precipitate was re-suspended with PBS, centrifuged at 110,000 ×*g* for 70 min, the precipitate was dissolved with 200 μL PBS, and stored at − 80 °C. Total exosome RNA and protein isolation kit (cat. no. 4478545; Invitrogen) was used to isolate RNA and proteins from exosomes and the protein levels of exosome surface markers CD9 (1:2000, ab92726, Abcam) and CD63 (1:2000, ab216130, Abcam) was measured by western blot.

#### Statistical analysis

All data were analyzed with SPSS 19.0 (IBM, Chicago, IL, USA) and illustrations were made with GraphPad Prism 8.0 (GraphPad Inc., LaJolla, California, USA). Pearson correlation analysis was used to analyze the expression association. Student’s *t*-test and one or two-way analysis of variance (ANOVA) were used to analyze differences between three or more groups. The results were shown as “mean ± standard deviation”. Statistical significance was identified as *P* value less than 0.05.

## Results

### The growth of HUVECs was suppressed by ox-LDL treatment

**Fig. 1:**
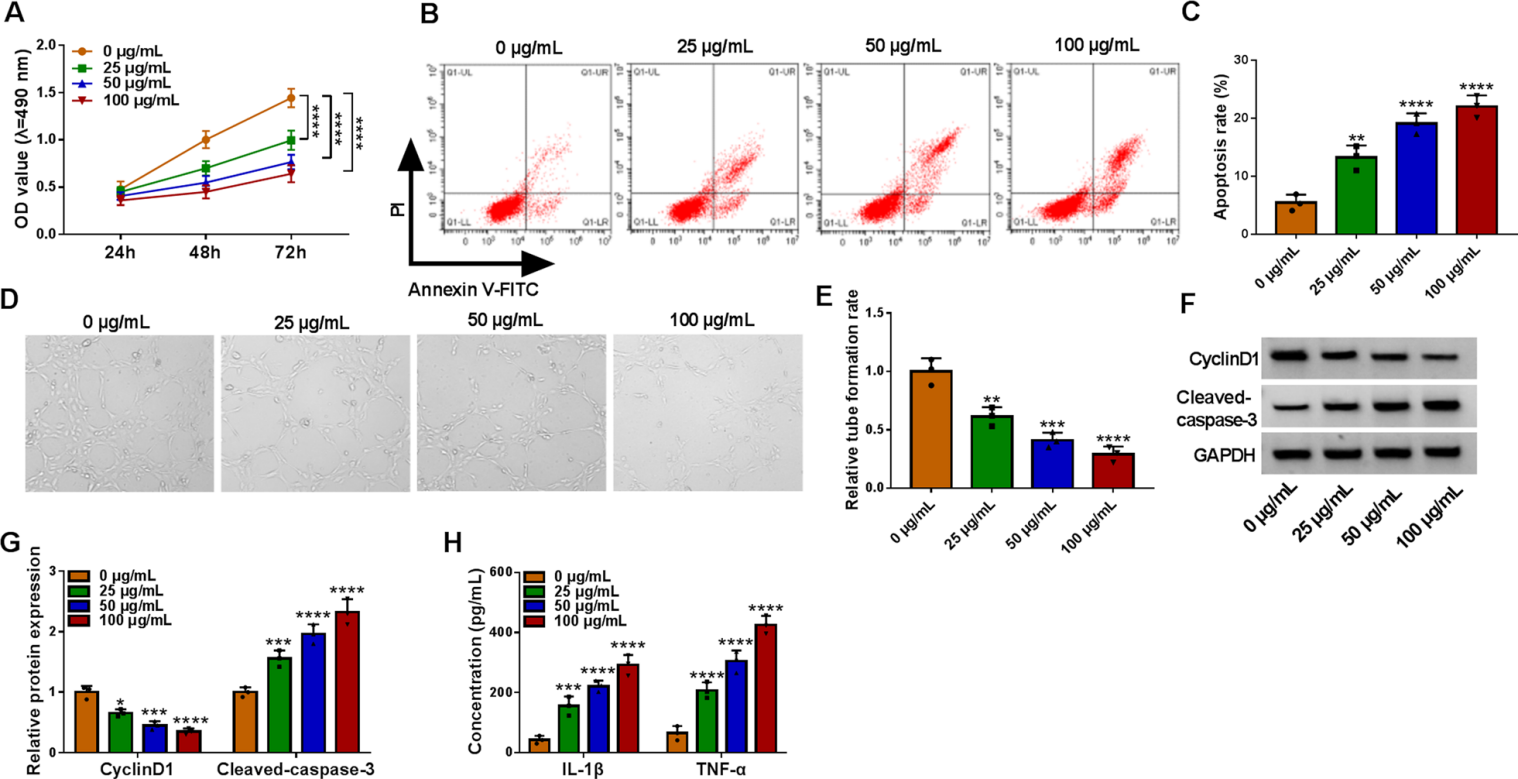
Ox-LDL impaired developments of HUVECs. HUVECs were treated with various doses of ox-LDL (0 μg/mL, 25 μg/mL, 50 μg/mL and 100 μg/mL) for 24 h. **A** MTT assay was used to detect cell viability in treated HUVECs. **B** and **C** Flow cytometry assay was performed to analyze the apoptosis rate in treated HUVECs. **D** and **E** Tube formation assay was conducted to determine the tube formation of HUVECs. **F** and **G** Western blot assay was applied to detect the protein levels of CyclinD1 and Cleaved-caspase-3 in treated HUVECs. **H** ELISA assay was used to measure the secretion of inflammatory factors IL-1β and TNF-α in treated HUVECs. *P < 0.05; **P < 0.01; ***P < 0.001; ****P < 0.0001. All tissue and cellular experiments were independently repeated three times. Two-way ANOVA was utilized to analyze the differences in (**A**, **G**, and **H**), while one-way ANOVA was utilized to assess the differences in (**B** and **D**)

The effects of ox-LDL on HUVECs were detected by treating cells with different concentrations of ox-LDL, and we found that the cell viability of HUVECs was significantly suppressed by ox-LDL in a concentration-dependent manner (Fig. 1A). On the contrary, ox-LDL promoted HUVECs cell apoptosis in a certain of concentration-dependent manner (Fig. 1B, C). For tube formation assay, compared with the 0 μg/mL group, angiogenesis gradually repressed in ox-LDL with the concentration of ox-LDL increase (Fig. 1D, E). And the protein expression of CyclinD1 was retarded by ox-LDL, while ox-LDL enhanced the protein level of Cleaved-caspase-3 in HUVECs in HUVECs after treatment of 0–100 μg/mL ox-LDL for 24 h (Fig. 1F, G). Moreover, ox-LDL increased the release of inflammatory factors IL-1β and TNF-α in HUVECs (Fig. 1H). In a word, ox-LDL blocked the progress of HUVECs in a dose-dependent manner.

### Knockdown of circ_0004104 promoted proliferation and suppressed apoptosis in ox-LDL-stimulated human endothelial cells

**Fig. 2:**
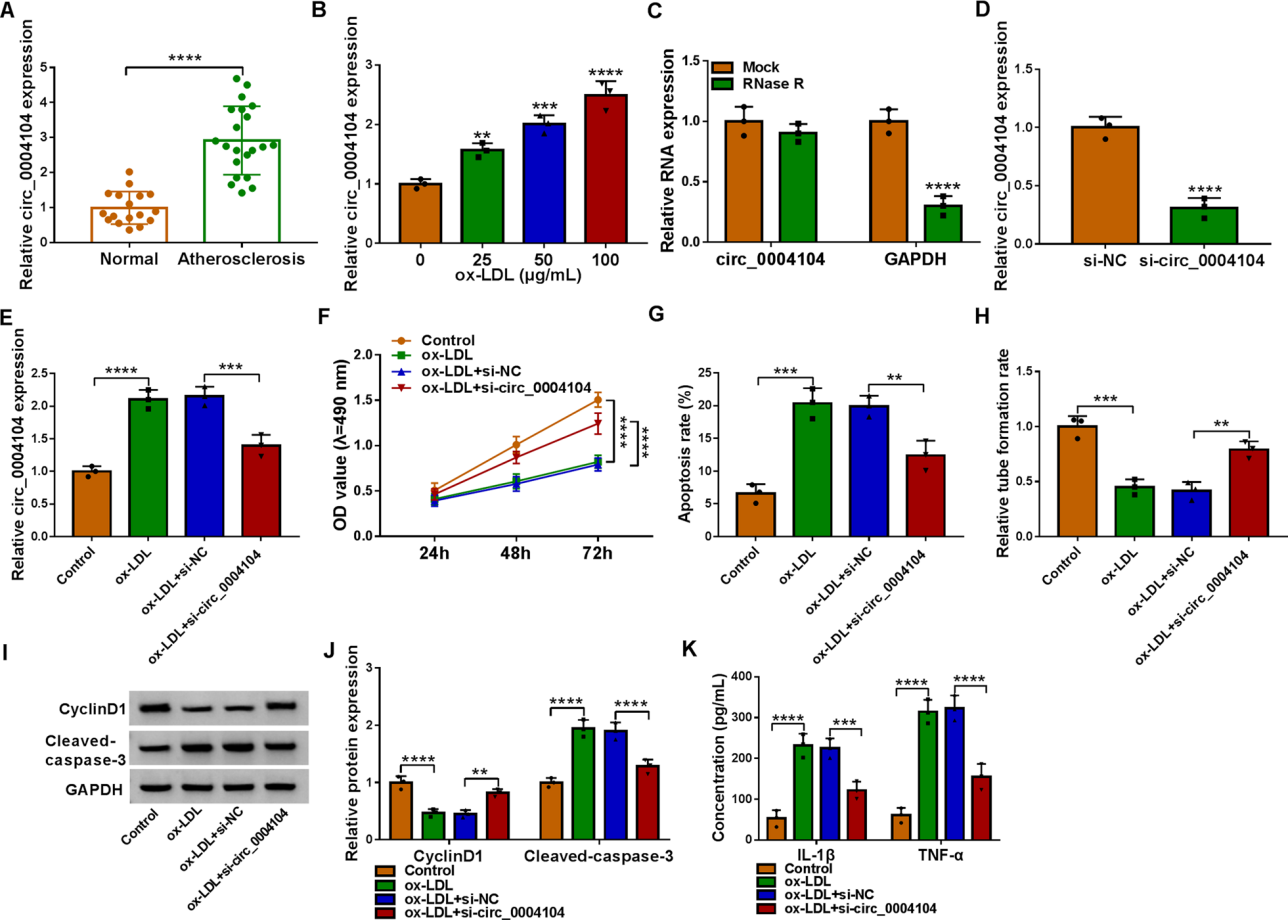
Circ_0004104 knockdown boosted proliferation and inhibited inflammatory in ox-LDL-exposed HUVECs. **A** Circ_0004104 level was determined in blood samples from 21 atherosclerosis patients and 17 healthy volunteers using qRT-PCR. **B** Circ_0004104 expression was measured in HUVECs treated with 0 μg/mL, 25 μg/mL, 50 μg/mL and 100 μg/mL of ox-LDL using qRT-PCR. **C** qRT-PCR analysis of circ_0004104 expression in HUVECs treated with or without RNase R assay. **D** qRT-PCR analysis of circ_0004104 expression HUVECs transfected with si-NC or si-circ_0004104. (**E**–**J**) HUVECs were treated with Control, ox-LDL, ox-LDL + si-NC, or ox-LDL + si-circ_0004104. **E** qRT-PCR analysis of circ_0004104 expression in treated HUVECs. **F** Cell proliferation was assessed in treated HUVECs using MTT assay. **G** Apoptosis rate was analyzed in treated HUVECs using flow cytometry assay. **H** The ability of HUVEC tube formation was assessed using tube formation assay. **I** and **J** Protein levels of CyclinD1 and Cleaved-caspase-3 in treated HUVECs were detected using western blot assay. **K** The secretion of inflammatory factors IL-1β and TNF-α in treated HUVECs was analyzed using ELISAs. **P < 0.01; ***P < 0.001; ****P < 0.0001. All cellular experiments were independently repeated three times. Student’s *t*-test was used to analyze the differences in (**A** and **D**), one-way ANOVA was utilized to assess the differences in (**F**, **I**, and **K**), and two-way ANOVA was utilized to analyze the differences in (**B**, **C**, **G**, and **H**)

The data of qRT-PCR disclosed that circ_0004104 was aberrantly augmented in serum samples from patients with atherosclerosis. (Fig. 2A). With the increase of ox-LDL dose, the expression of circ_0004104 in HUVECs also showed an increasing trend (Fig. 2B), and 50 μg/mL ox-LDL was selected for further research. The expression of linear GAPDH was obviously reduced in HUVECs treated with RNase R, while the level of circ_0004104 was not notably changed (Fig. 2C). In addition, our data displayed that the introduction of si-circ_0004104 might obviously reduce the expression of circ_0004104 in HUVECs, suggesting the knockdown efficiency of circ_0004104 is successful (Fig. 2D). And silencing circ_0004104 attenuated the promoting effect of ox-LDL on circ_0004104 expression (Fig. 2E). Ox-LDL exposure blocked cell viability, which was largely counteracted by circ_0004104 silencing in HUVECs (Fig. 2F). Cell apoptosis was triggered by ox-LDL exposure, and the interference of circ_0004104 restrained the apoptosis of ox-LDL-induced HUVECs (Fig. 2G). The tube formation rate in HUVECs was hindered by ox-LDL stimulation, and si-circ_0004104 enhanced the tube formation (Fig. 2H). Then we measured the protein levels of CyclinD1 and Cleaved-caspase-3 in HUVECs, circ_0004104 downregulation rescued ox-LDL-induced changes in CyclinD1 and Cleaved-caspase-3 protein levels (Fig. 2I, J). Silence of circ_0004104 weakened the ascending effects of ox-LDL on IL-1β and TNF-α expression in HUVECs (Fig. 2K). These data suggested that circ_0004104 downregulation might relieve ox-LDL-triggered cell injury.

### Circ_0004104 directly interacted with miR-942-5p

**Fig. 3:**
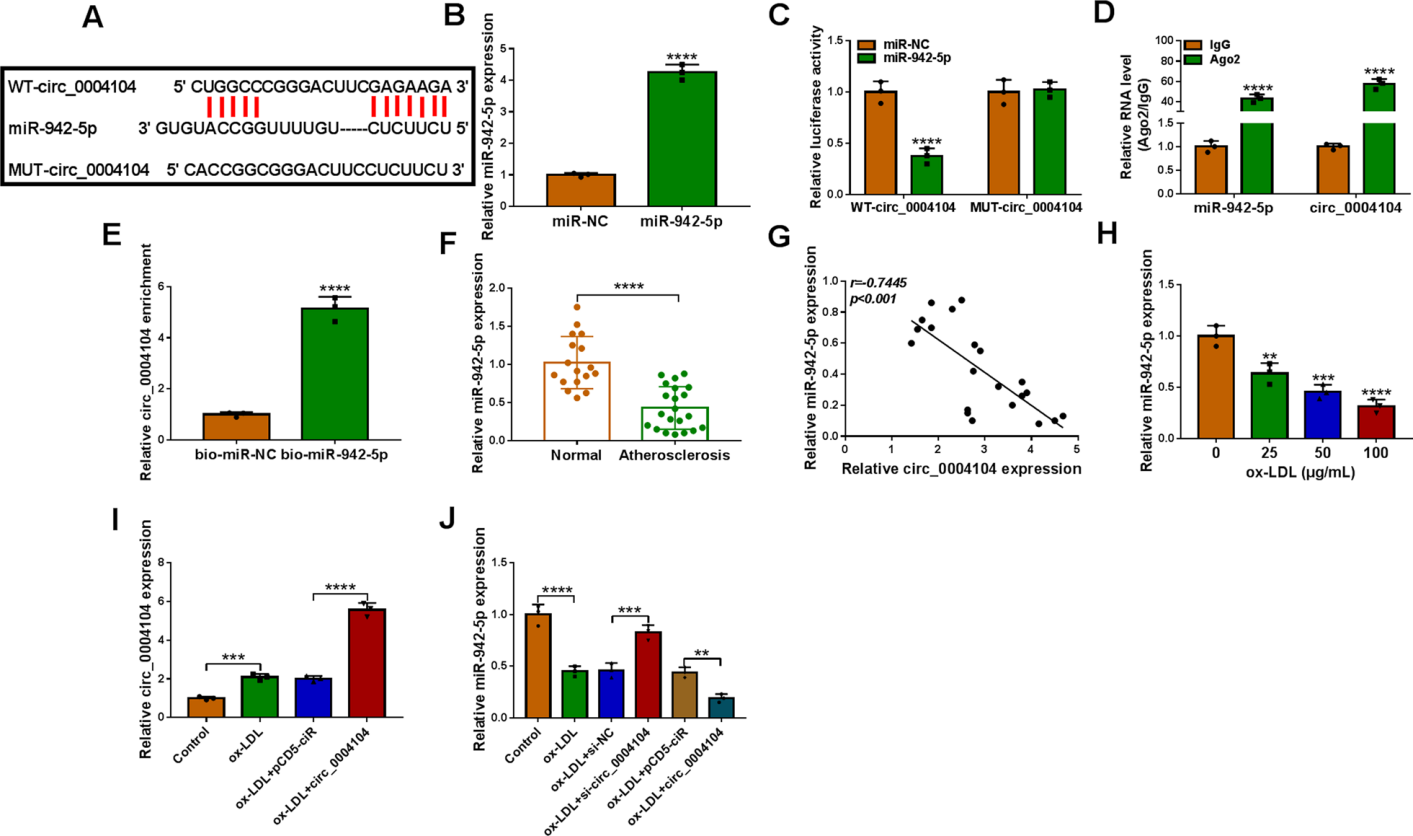
Circ_0004104 acted as a sponge of miR-942-5p. **A** The binding sites between circ_0004104 and miR-942-5p were shown. **B** MiR-942-5p expression in HUVECs transfected with miR-942-5p mimics using qRT-PCR. **C** The luciferase activities of WT-circ_0004104 and MUT-circ_0004104. **D** RIP assay was performed to analyze the enrichment of circ_0004104 and miR-942-5p. **E** Their interaction was verified using RNA pull-down assay. **F** qRT-PCR analysis of miR-942-5p in serum samples from 21 patients with atherosclerosis and 17 healthy volunteers. **G** Pearson correlation analysis was applied to evaluate the expression association between circ_0004104 and miR-942-5p in 21 atherosclerosis patients. **H** qRT-PCR analysis of miR-942-5p in HUVECs treated with 0 μg/mL, 25 μg/mL, 50 μg/mL and 100 μg/mL of ox-LDL. **I** qRT-PCR analysis of circ_0004104 in HUVECs treated with Control, ox-LDL, ox-LDL + pCD5-ciR, and ox-LDL + circ_0004104. **J** qRT-PCR analysis of miR-942-5p in HUVECs treated with Control, ox-LDL, ox-LDL + si-NC, ox-LDL + si-circ_0004104, ox-LDL + pCD5-ciR, or ox-LDL + circ_0004104. **P < 0.01; ***P < 0.001; ****P < 0.0001. All tissue and cellular experiments were independently repeated three times. Student’s *t*-test was used to analyze the differences in (**B**, **E**, and **F**), one-way ANOVA was utilized to assess the differences in (**H**, **I**, and **J**), and two-way ANOVA was utilized to analyze the differences in (**C**, **D**)

The targets of circ_0004104 were forecasted by Circinteractome (https://circinteractome.nia.nih.gov), and the data indicated that miR-942-5p could bind to circ_0004104, as illustrated in Fig. 3A. The transfection efficiency of miR-942-5p mimics was detected by qRT-PCR, and the consequence exhibited that the expression of miR-942-5p in HUVECs was high after transfected with miR-942-5p mimics (Fig. 3B). The data of dual-luciferase reporter assay uncovered that miR-942-5p hampered the luciferase activity of WT-circ_0004104 in HUVECs, while there was no significantly changes of luciferase activity in MUT-circ_0004104 (Fig. 3C). Then we found that circ_0004104 and miR-942-5p were more abundant in Ago2 pellet than in the IgG pellet in HUVECs (Fig. 3D). Meanwhile, RNA pull-down assay displayed that the enrichment of circ_0004104 in the captured fraction was prominently improved in the bio-miR-942-5p group compared with the bio-miR-NC group (Fig. 3E). QRT-PCR assay uncovered the expression of miR-942-5p was greatly reduced in serum samples of Atherosclerosis patients (Fig. 3F). And the Pearson correlation analyzed hinted that the expression of miR-942-5p in Atherosclerosis patients was passively related to circ_0004104 level (Fig. 3G). Importantly, miR-942-5p level in HUVECs was gradually decreased in HUVECs after treatment of 0–100 μg/mL ox-LDL (Fig. 3H). We found that circ_0004104 overexpression remarkably boosted the promoting effect of ox-LDL stimulation on circ_0004104 content in HUVECs (Fig. 3I). The inhibitory effects of ox-LDL on miR-942-5p level in HUVECs were overturned by circ_0004104 downregulation; while overexpression of circ_0004104 enhanced the inhibitory impact of ox-LDL on miR-942-5p expression (Fig. 3J). Based on the results above, we concluded that circ_0004104 might function as a competing endogenous RNA (ceRNA) by targeting miR-942-5p.

### Circ_0004104 downregulation participated in the regulation of ox-LDL-induced HUVECs injury via targeting miR-942-5p

**Fig. 4:**
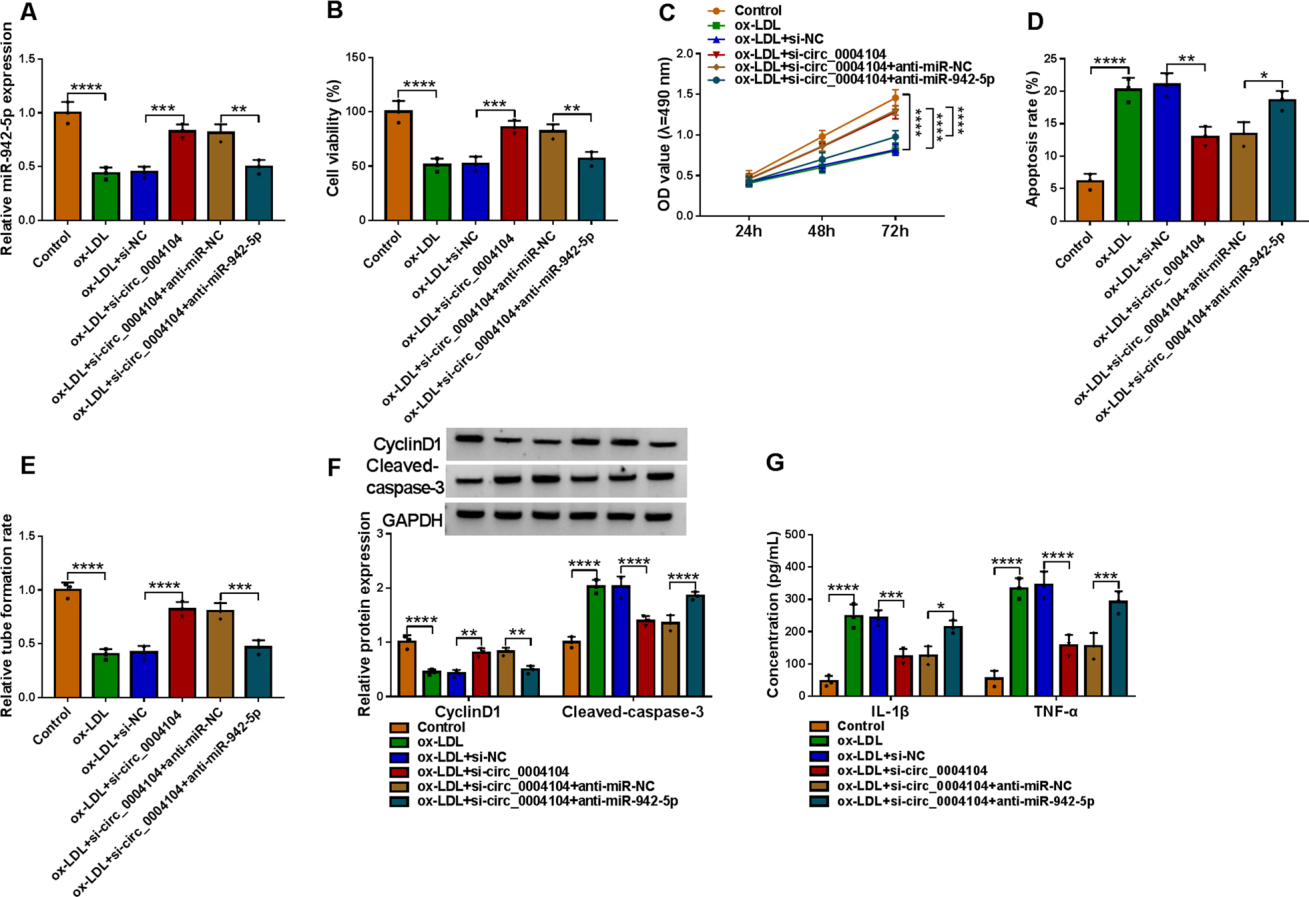
Circ_0004104 regulated the progression of HUVECs through targeting miR-942-5p. HUVECs were treated with Control, ox-LDL, ox-LDL + si-NC, ox-LDL + si-circ_0004104, ox-LDL + si-circ_0004104 + anti-miR-NC, or ox-LDL + si-circ_0004104 + anti-miR-942-5p. **A** qRT-PCR analysis of miR-942-5p in treated HUVECs. **B**–**G** Cell viability, apoptosis, tube formation, protein expression levels, and inflammatory factors in treated HUVECs were detected using MTT, flow cytometry, tube formation, western blot, and ELISA assays. *P < 0.05; **P < 0.01; ***P < 0.001; ****P < 0.0001. All cellular experiments were independently repeated three times. One-way ANOVA was utilized to analyze the differences in (**A**, **B**, **D**, and **E**), while two-way ANOVA was utilized to assess the differences in (**C**, **F**, and **G**)

As shown in Fig. 4A, si-circ_0004104 triggered the expression of miR-942-5p in HUVECs exposed to ox-LDL, while the effect was abated by anti-miR-942-5p. Results of MTT in Fig. 4B, C displayed that silencing the expression of circ_0004104 effectively promoted cell viability of HUVECs treated with ox-LDL, while the loss of miR-942-5p evidently retarded the effects of si-circ_0004104 on cell viability. After HUVECs were treated with ox-LDL, circ_0004104 silencing retarded HUVECs apoptosis, whereas co-transfection of anti-miR-942-5p abrogated this impact in part (Fig. 4D). About the tube formation rate of HUVECs under ox-LDL, si-circ_0004104 and anti-miR-942-5p treatments showed the same trend as that of proliferation in Fig. 4C (Fig. 4E). Besides, circ_0004104 knockdown elevated CyclinD1 level in ox-LDL-disposed HUVECs, while this impact was partly counteracted by anti-miR-942-5p, and the expression trend of Cleaved-caspase-3 was opposite to CyclinD1 (Fig. 4F). The addition of miR-942-5p inhibitor to ox-LDL-stimulated HUVECs restored the effects of down-regulated circ_0004104 on IL-1β and TNF-α contents (Fig. 4G). Collectively, these results implied that miR-942-5p downregulation might overturn the repression of circ_0004104 deficiency on ox-LDL-induced cell damage.

### MiR-942-5p directly interacted with ROCK2

**Fig. 5:**
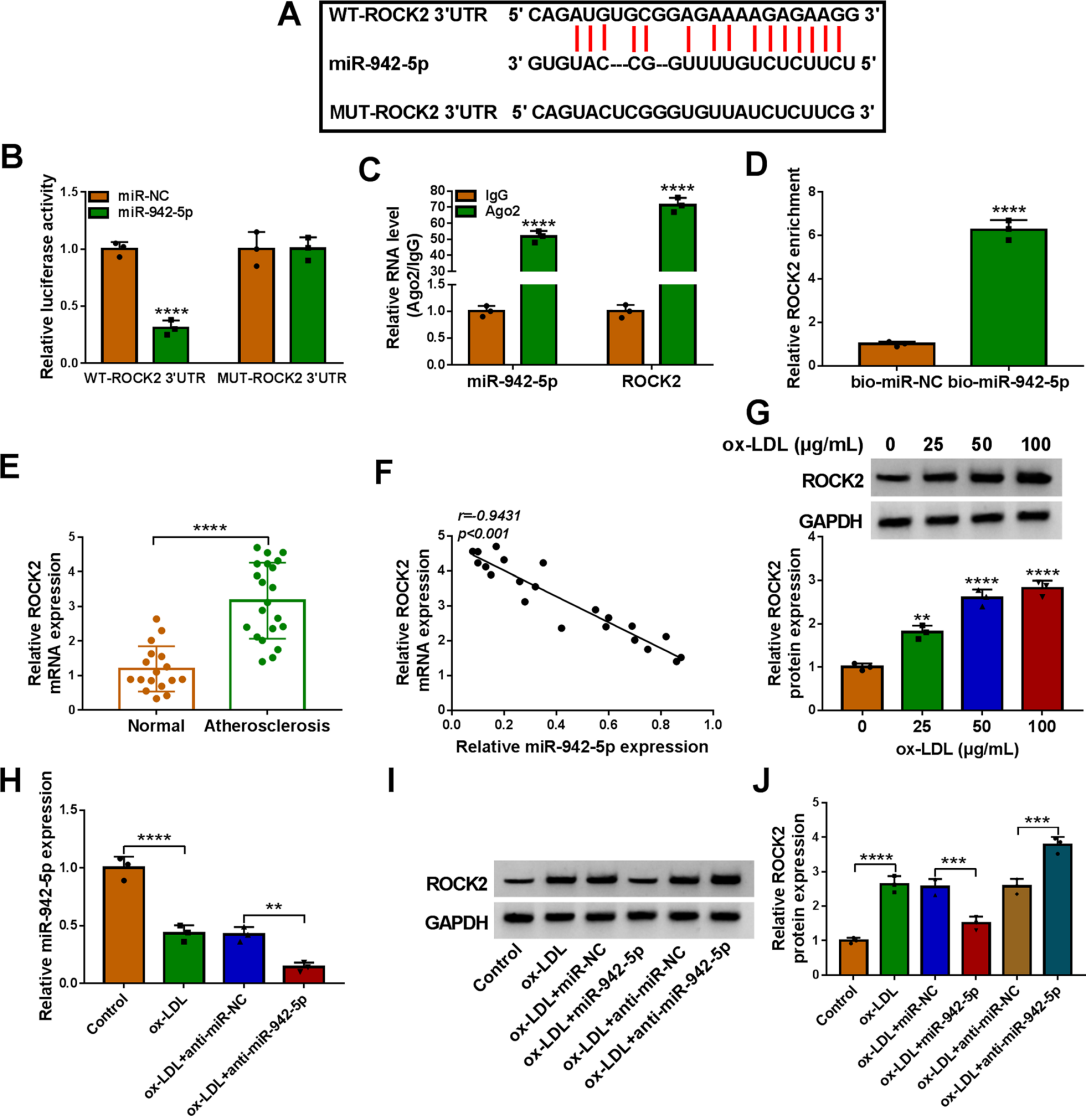
ROCK2 was regulated by miR-942-5p. **A** The binding sites of miR-942-5p and ROCK2 were displayed. **B**–**D** The relationship between ROCK2 and miR-942-5p was assessed by dual-luciferase reporter assay, RIP assay, and RNA pull-down assay. **E** ROCK2 level was assessed in serum samples from 21 patients with atherosclerosis and 17 healthy volunteers using qRT-PCR. **F** The expression correlation between ROCK2 and miR-942-5p in 21 atherosclerosis tissues was analyzed by Pearson correlation analysis. **G** The protein expression of ROCK2 in ox-LDL-treated HUVECs was determined using western blot assay. **H** qRT-PCR analysis of miR-942-5p in HUVECs treated with Control, ox-LDL, ox-LDL + anti-miR-NC, or ox-LDL + anti-miR-942-5p. **I** and **J** ROCK2 protein expression was assessed in HUVECs treated with Control, ox-LDL, ox-LDL + miR-NC, ox-LDL + miR-942-5p, ox-LDL + anti-miR-NC, or ox-LDL + anti-miR-942-5p using western blot assay. **P < 0.01; ***P < 0.001; ****P < 0.0001. All tissue and cellular experiments were independently repeated three times. Student’s *t*-test was used to analyze the differences in (**D**, **E**), one-way ANOVA was utilized to assess the differences in (**G**, **H**, and **I**), and two-way ANOVA was utilized to analyze the differences in (**B**, **C**)

According to the prediction of Starbase (http://starbase.sysu.edu.cn/), ROCK2 3’UTR had a binding region with miR-942-5p (Fig. 5A), and the target effect between ROCK2 and miR-942-5p was ascertained by dual-luciferase reporter assay, RIP assay, and RNA pull-down assay (Fig. 5B–D). Then we probed the expression of ROCK2 in Atherosclerosis patients, as depicted in Fig. 5E, ROCK2 mRNA was boosted in serum samples of Atherosclerosis patients. And the expression of ROCK2 was negatively correlated with the level of miR-942-5p in Atherosclerosis patients (Fig. 5F). Our data suggested that the protein expression of ROCK2 in ox-LDL-induced HUVECs was exceptionally intensified in a dose-dependent manner (Fig. 5G). And miR-942-5p depletion deepened the inhibition of ox-LDL on miR-942-5p expression in HUVECs (Fig. 5H). Finally, the results of western blot exhibited that miR-942-5p upregulation distinctly repressed ROCK2 expression in HUVECs under ox-LDL treatment and inhibition of miR-942-5p accelerated the promotion of ROCK2 expression by ox-LDL stimulation in HUVECs (Fig. 5I, J). Taken together, these data indicated that ROCK2 acted as a target of miR-942-5p.

### MiR-942-5p affected the cell process by affecting the expression of ROCK2

**Fig. 6:**
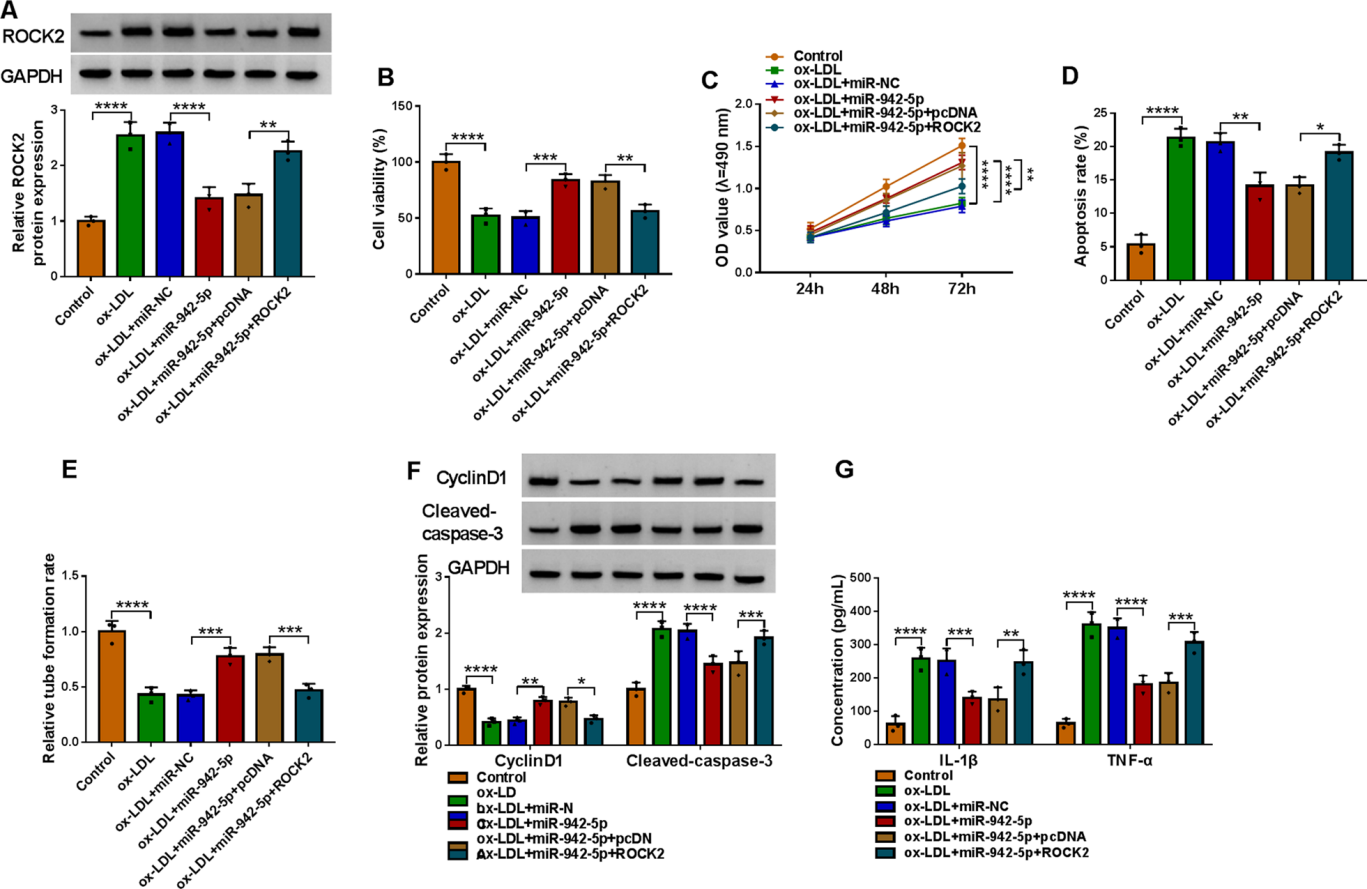
ROCK2 overexpression overturned miR-942-5p-mediated functions in cell viability, proliferation, apoptosis, angiogenesis, and inflammatory response. HUVECs were treated with Control, ox-LDL, ox-LDL + miR-NC, ox-LDL + miR-942-5p, ox-LDL + miR-942-5p + pcDNA, or ox-LDL + miR-942-5p + ROCK2. **A** Western blot analysis of ROCK2 in treated HUVECs. **B**–**G** Cell viability, apoptosis, tube formation, protein expression levels, and inflammatory factors in treated HUVECs were analyzed by MTT, flow cytometry, tube formation, western blot, and ELISA assays. *P < 0.05; **P < 0.01; ***P < 0.001; ****P < 0.0001. All cellular experiments were independently repeated three times. One-way ANOVA was utilized to analyze the differences in (**A**, **B**, **D**, and **E**), while two-way ANOVA was utilized to assess the differences in (**C**, **F**, and **G**)

The repression effect of miR-942-5p on ROCK2 in ox-LDL-induced HUVECs was recovered by the reintroduction of ROCK2 (Fig. 6A). Overexpression of miR-942-5p obviously contributed to cell viability, but blocked cell apoptosis of ox-LDL-exposed HUVECs, which were rescued by additional ROCK2 (Fig. 6B–D). In HUVECs treated with ox-LDL, ROCK2 upregulation overturned tube formation that was aggrandized by miR-942-5p transfection (Fig. 6E). After HUVECs treated with ox-LDL, the level of CyclinD1 in HUVECs transfected with miR-942-5p was enhanced, while it was decreased in HUVECs co-transfected with ROCK2 and miR-942-5p, but the levels of Cleaved-caspase-3 were inverse to the level of CyclinD1 (Fig. 6F). Furthermore, the secretion of IL-1β and TNF-α was inhibited in ox-LDL-treated HUVECs transfected with miR-942-5p, and these effects were abolished after miR-942-5p and ROCK2 were co-transfected (Fig. 6G). Collectively, these results indicated that miR-942-5p might repress ox-LDL-aroused cell injury by regulating ROCK2.

### Circ_0004104 regulated ROCK2 expression by sponging miR-942-5p

**Fig. 7:**
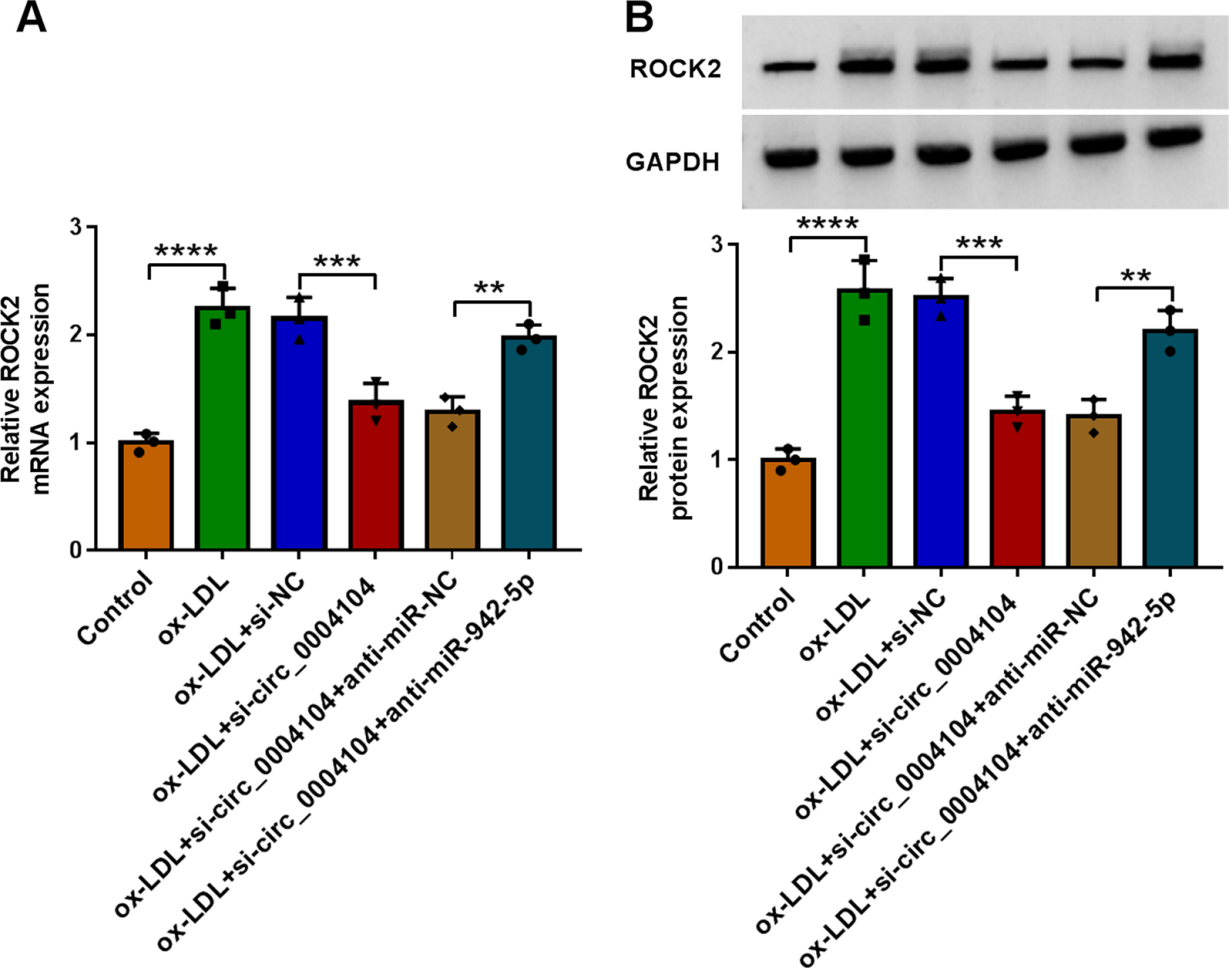
miR-942-5p downregulation abolished the inhibitory role of circ_0004104 silencing on ROCK2 expression in ox-LDL-exposed HUVECs. **A** and **B** ROCK2 expression was detected in HUVECs treated with Control, ox-LDL, ox-LDL + si-NC, ox-LDL + si-circ_0004104, ox-LDL + si-circ_0004104 + anti-miR-NC, or ox-LDL + si-circ_0004104 + anti-miR-942-5p using qRT-PCR and western blot assay. *P < 0.05; **P < 0.01; ***P < 0.001; ****P < 0.0001. All cellular experiments were independently repeated three times. One-way ANOVA was utilized to analyze the differences in all results in this figure

The data of qRT-PCR and western blot assays displayed that circ_0004104 downregulation inhibited the levels of ROCK2 in HUVECs treated with ox-LDL, and these effects were fractionally counteracted after anti-miR-942-5p was co-transfected (Fig. 7A, B). Overall, these results indicated that circ_0004104 served as a molecular sponge to sequester miR-942-5p from ROCK2.

### Exosomes were involved in mediating delivery of circ_0004104

**Fig. 8:**
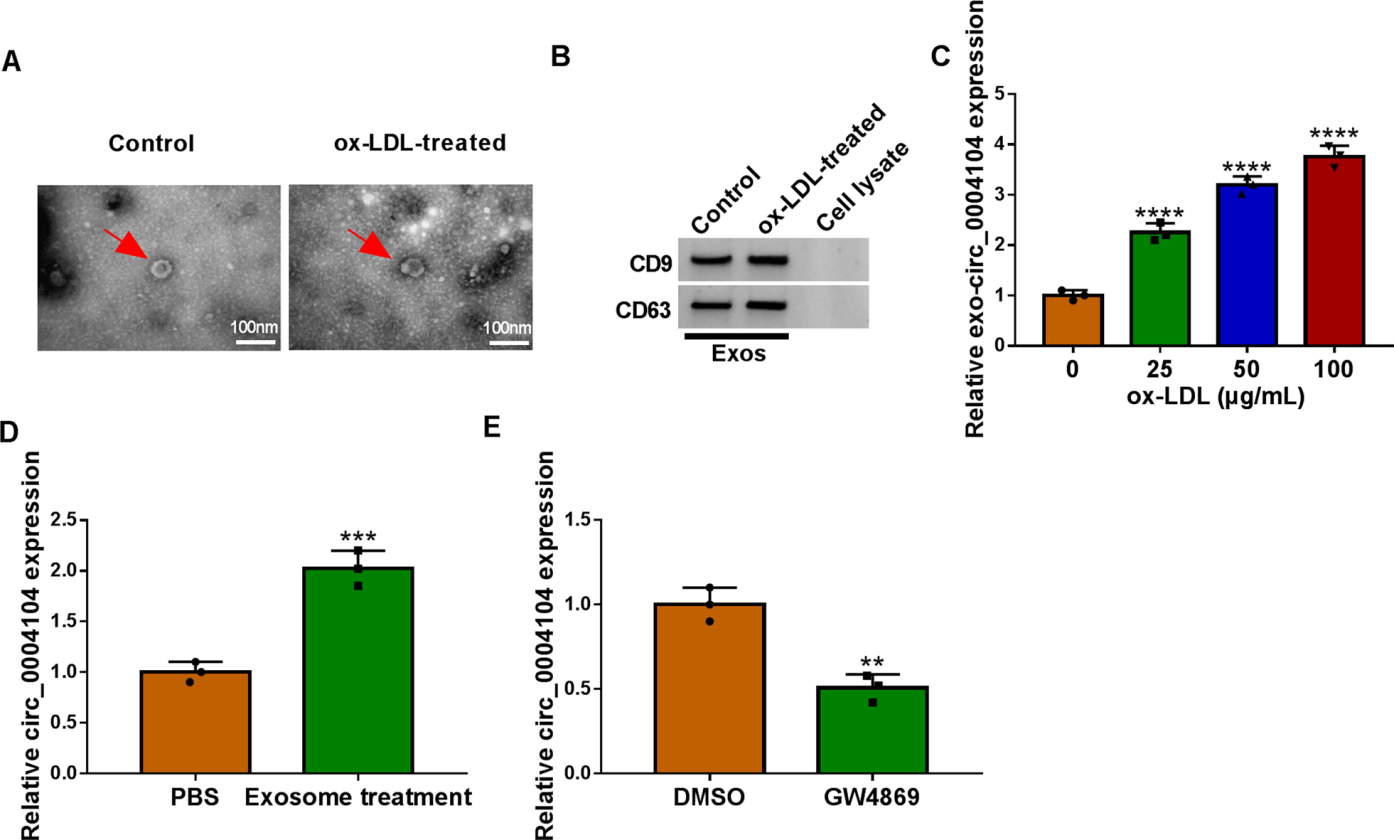
Ox-LDL exposure to HUVECs-derived exosomes regulated the expression of circ_0004104. **A** Image of exosomes was observed under a transmission electron microscope. **B** Detection of exosomal markers was performed using western blot assay. **C** Circ_0004104 expression levels in exosomes treated with different concentrations of ox-LDL were detected using qRT-PCR assay. **D** Expression levels of circ_0004104 were detected using qRT-PCR assay in HUVECs treated with exosomes. **E** Expression levels of circ_0004104 in HUVECs treated with exosome isolation inhibitor (GW4869) and DMSO were detected using qRT-PCR assay. **P < 0.01; ***P < 0.001; ****P < 0.0001. All cellular experiments were independently repeated three times. Student’s *t*-test was used to analyze the differences in (**D**, **E**), whereas one-way ANOVA was utilized to assess the differences in (**C**)

HUVECs were divided into two groups, the ox-LDL treatment group was the experimental group, while the untreated group was the control group. Extracellular exosomes were extracted from HUVECs of the two groups and confirmed by transmission electron microscopy and western blot. Extracellular exosomes displayed spherical structures with a diameter of approximately 100 nm (Fig. 8A). As shown in Fig. 8B, the proteins of CD9 and CD63 were positively expressed in extracellular exosomes derived from the two groups. The results of Fig. 8C demonstrated that the expression of circ_0004104 in ox-LDL treated extracellular exosomes was significantly augmented in a concentration-dependent manner. Then we incubated HUVECs with exosomes extracted from the ox-LDL-treatment group and replaced exosomes incubated HUVECs with PBS as the control group, and the expression of circ_0004104 in groups was detected, as presented in Fig. 8D, circ_0004104 in Exosome treatment group was obviously boosted than PBS group. Importantly, the expression of circ_0004104 in the cell culture medium of the exosome inhibitor GW4869 treatment group was lower than that in the DMSO group (Fig. 8E). In summary, these data suggested that delivery of circ_0004104 might be mediated by exosome.

## Discussion

Atherosclerosis was characterized by thickening and hardening of the arterial wall, loss of elasticity, and narrowing of the lumen. It was related to endothelial damage [25], inflammation, and immunity [26], and was easy to induce coronary heart disease, cerebral infarction, and peripheral vascular disease [27]. Therefore, it is urgent to explore the development of atherosclerosis. Our study explored the function and mechanism of circ_0004104, miR-942-5p, and ROCK2 in atherosclerosis through *in vitro* and *in vivo* experiments, providing new biomarkers for the treatment of atherosclerosis.

Studies have shown that ox-LDL promoted plaque formation and played a key role in atherosclerosis [28]. Therefore, in this study, HUVECs were induced with ox-LDL to construct atherosclerosis models and the effects of ox-LDL on HUVECs viability, proliferation, apoptosis, and inflammatory response were detected. The results revealed that ox-LDL inhibited the proliferation of HUVECs and induced cell apoptosis in a concentration-dependent manner. Moreover, ox-LDL accelerated the release of inflammatory cytokines IL-1β and TNF-α in HUVECs, indicating that ox-LDL caused HUVEC damage. In addition, Gao *et al*. implied that circ_0004104 might be involved in cardiovascular disease through bioinformatics tools [11]. And we confirmed that circ_0004104 was enriched in atherosclerosis patients and models and si-circ_0004104 reduced the harm of ox-LDL to HUVECs. These results were consistent with those of Zhang *et al* [29], they suggested that ectopic expression of circ_0004104 restored the effects of ox-LDL on HUVECs. We also suggested that silencing circ_0004104 attenuated ox-LDL-induced inflammatory response in HUVECs. Further research disclosed that ox-LDL treatment promoted the expression of circ_0004104 in exosomes. After exosome treatment, the content of circ_0004104 in HUVECs increased, while exosome secretion inhibitor GW4869 attenuated the release of circ_0004104 in HUVECs. These results demonstrated the protective effects of circ_0004104 knockdown on ox-LDL-exposed HUVECs.

The spongy role between miRNAs and circRNAs had been demonstrated [30, 31], for instance, circ_0093887 sponged miR-876-3p and miR-876 to protect human aortic endothelial cells (HAECs) from ox-LDL-induced damage [32]; Silencing circ_GRN acted as a sponge of miR-214-3p to abolish migration, proliferation, and inflammation of ox-LDL-exposed VSMCs [33]. In our study, we found that circ_0004104 sponged miR-942-5p in atherosclerosis. Previously, miR-942-5p was reported to be hindered by ox-LDL treatment in HUVECs [19], and in our current study, miR-942-5p was negatively adjusted by circ_0004104 and was decreased in the serum of Atherosclerosis patients and ox-LDL-stimulated HUVECs. Furthermore, miR-942-5p downregulation attenuated the effects of si-circ_0004104 in ox-LDL-provoked damage of HUVECs. Our research was the first to demonstrate the sponge role of circ_0004104 and miR-942-5p in atherosclerosis.

It was previously found that ROCK2 was obviously increased in HUVECs treated with ox-LDL and atherosclerosis patients [23, 34]. Analogously, our observations also indicated that ROCK2 was notably upregulated in atherosclerosis patients, and ox-LDL treatment boosted ROCK2 level in HUVECs in a concentration-dependent manner. In addition, Miao et al. [34] discovered that ROCK2 inhibition promoted migration and invasion and confined apoptosis and inflammation in ox-LDL-treated HUVECs. Our data proved that ROCK2 was mediated by miR-942-5p and circ_0004104, and miR-942-5p overexpression mitigated the damage of ox-LDL to HUVECs, while the introduction of ROCK2 weakened the influences of miR-942-5p in part. All these displayed that circ_0004104 attenuated ox-LDL-induced damage to HUVECs through miR-942-5p and ROCK2.

## Conclusion

**Fig. 9:**
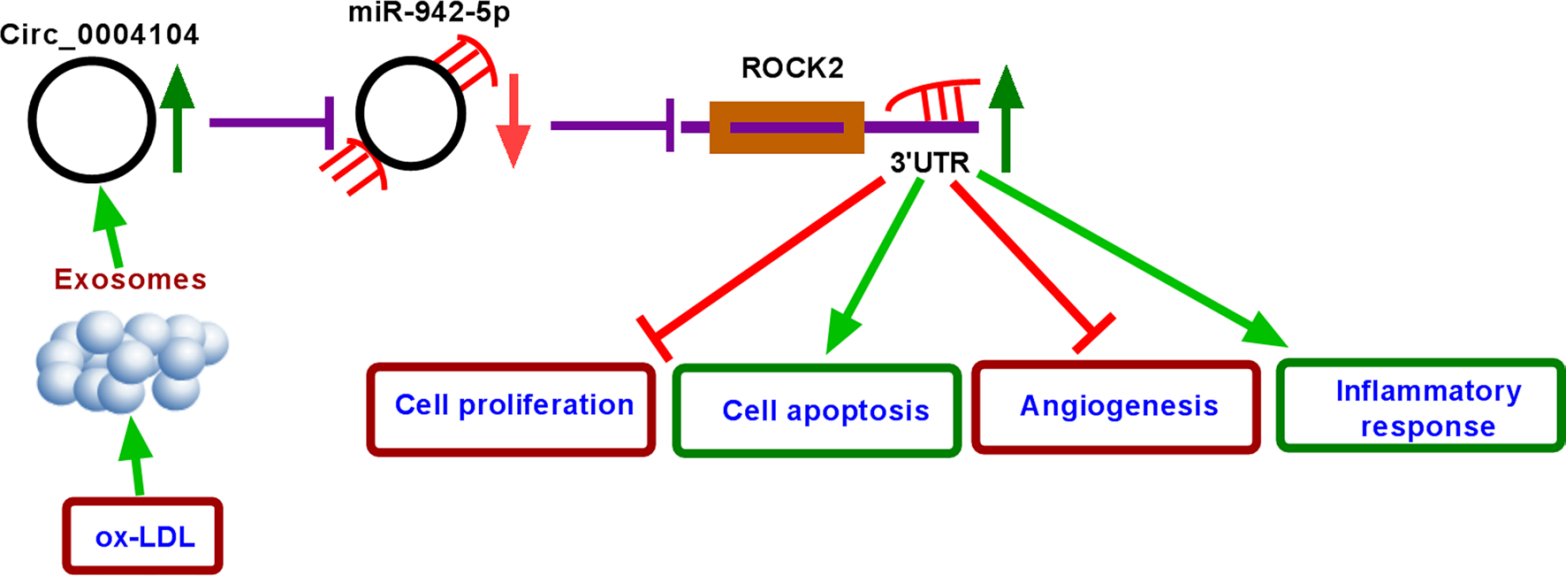
circ_0004104 knockdown promoted the proliferation and inhibited inflammatory reaction in ox-LDL-exposed HUVECs through the miR-942-5p/ROCK2 axis

In conclusion, our data manifested that ox-LDL induced the cell injury of HUVECs, while circ_0004104 knockdown promoted the proliferation and inhibited inflammatory reaction in ox-LDL-exposed HUVECs through the miR-942-5p/ROCK2 axis (Fig. 9). The circ_0004104/miR-942-5p/ROCK2 regulatory pathway might provide a new direction for the treatment of atherosclerosis.

## Supplementary Information

**Additional file 1:** The original western blots.

## Data Availability

The data sets used and/or analyzed during the current study are available from the corresponding author on reasonable request.
