# Supplementary material for: Circ_0004104 participates in the regulation of ox-LDL-induced endothelial cells injury via miR-942-5p/ROCK2 axis
Source: BMC Cardiovasc Disord. 2022 Dec 2;22:517. doi: 10.1186/s12872-022-02959-1 (PMC9717494; doi:10.1186/s12872-022-02959-1)
Supplement: Supplementary file 1 — Additional file 1: The original western blots. [file 12872_2022_2959_MOESM1_ESM.pdf]

The original western blots of Fig1

F

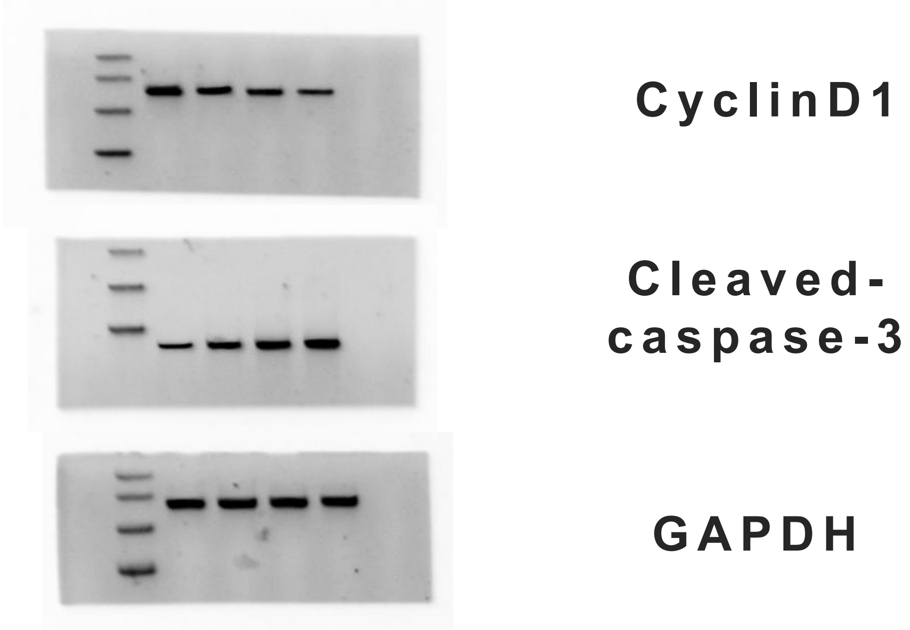

The original western blots of Fig2

I

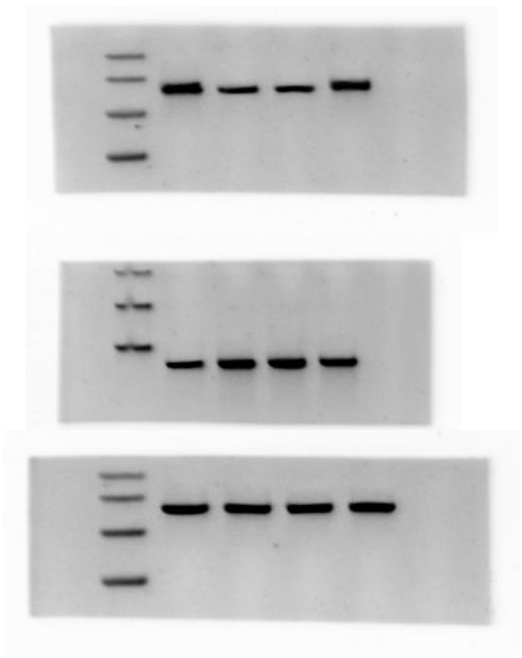

The original western blots of Fig4

F

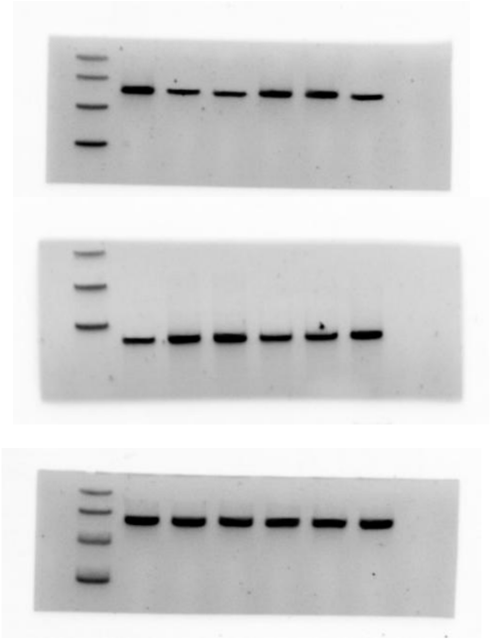

CyclinD1

Cleaved-
caspase-3

GAPDH

The original western blots of Fig5

G

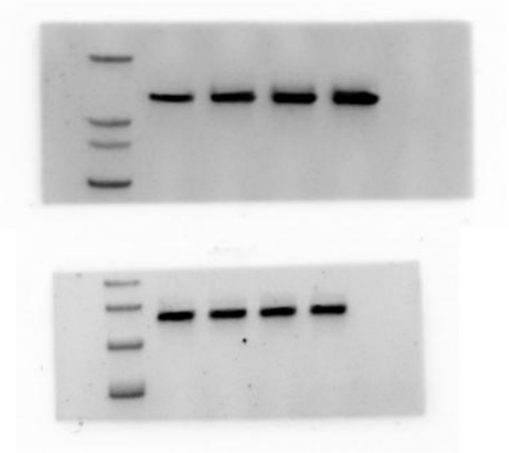

ROCK2

GAPDH

I

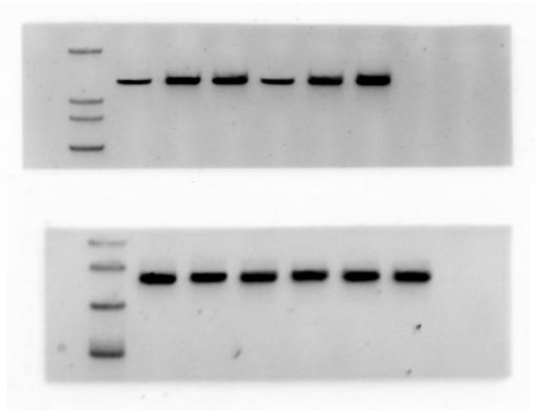

ROCK2

GAPDH

The original western blots of Fig6

A

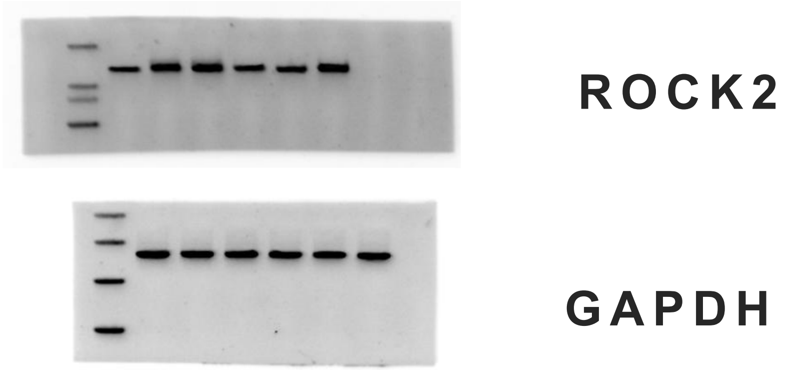

The original western blots of Fig6

F

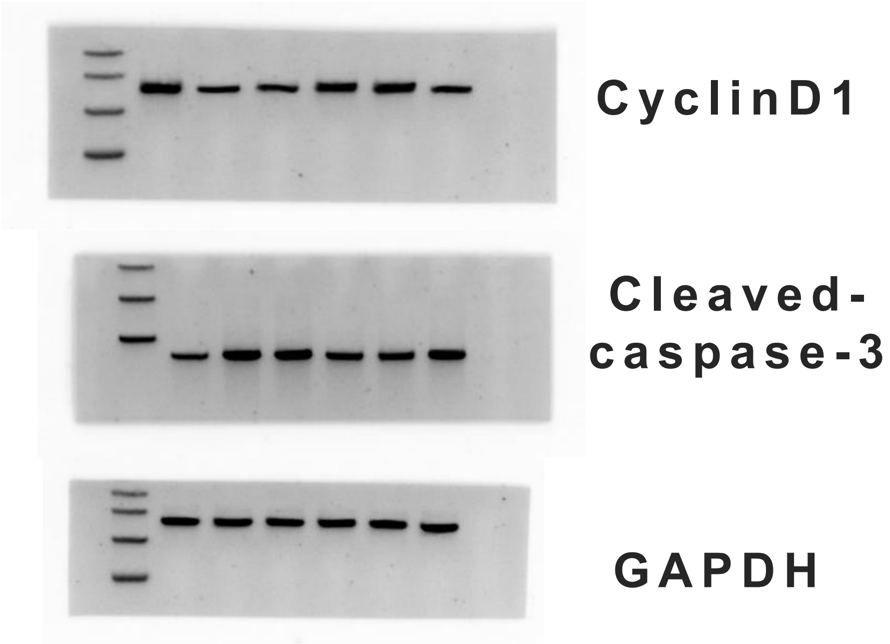

The original western blots of Fig7

B

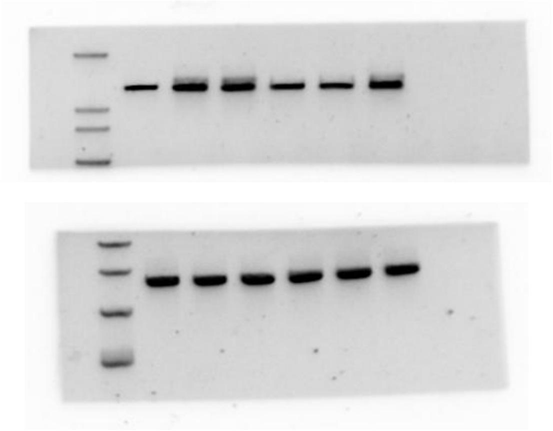

ROCK2

GAPDH

The original western blots of Fig8

B

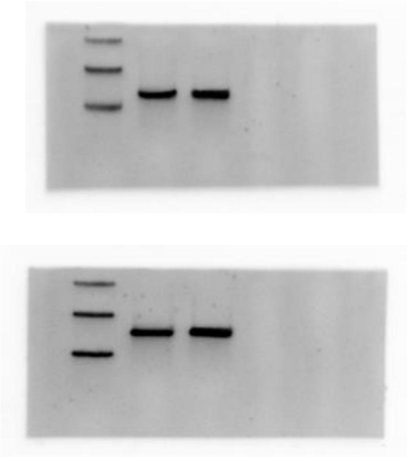

ROCK2

GAPDH
